# Engineering the xylose‐catabolizing Dahms pathway for production of poly(d‐lactate‐*co*‐glycolate) and poly(d‐lactate‐*co*‐glycolate‐*co*‐d‐2‐hydroxybutyrate) in *Escherichia coli*

**DOI:** 10.1111/1751-7915.12721

**Published:** 2017-04-19

**Authors:** So Young Choi, Won Jun Kim, Seung Jung Yu, Si Jae Park, Sung Gap Im, Sang Yup Lee

**Affiliations:** ^1^ Metabolic and Biomolecular Engineering National Research Laboratory Department of Chemical and Biomolecular Engineering (BK21 Plus Program) BioProcess Engineering Research Center, and KAIST Institute (KI) for the BioCentury Korea Advanced Institute of Science and Technology (KAIST) 291 Daehak‐ro Yuseong‐gu Daejeon 34141 Korea; ^2^ Department of Chemical and Biomolecular Engineering (BK21 Plus Program) KAIST 291 Daehak‐ro Yuseong‐gu Daejeon 34141 Korea; ^3^ Department of Chemical Engineering and Materials Science Ewha Womans University 52 Ewhayeodae‐gil Seodaemun‐gu Seoul 03760 Korea

## Abstract

Poly(lactate‐*co*‐glycolate), PLGA, is a representative synthetic biopolymer widely used in medical applications. Recently, we reported one‐step direct fermentative production of PLGA and its copolymers by metabolically engineered *Escherichia coli* from xylose and glucose. In this study, we report development of metabolically engineered *E. coli* strains for the production of PLGA and poly(d‐lactate‐*co*‐glycolate‐*co*‐d‐2‐hydroxybutyrate) having various monomer compositions from xylose as a sole carbon source. To achieve this, the metabolic flux towards Dahms pathway was modulated using five different synthetic promoters for the expression of *Caulobacter crescentus* XylBC. Further metabolic engineering to concentrate the metabolic flux towards d‐lactate and glycolate resulted in production of PLGA and poly(d‐lactate‐*co*‐glycolate‐*co*‐d‐2‐hydroxybutyrate) with various monomer fractions from xylose. The engineered *E. coli* strains produced polymers containing 8.8–60.9 mol% of glycolate up to 6.93 g l^−1^ by fed‐batch cultivation in a chemically defined medium containing xylose. Finally, the biocompatibility of poly(d‐lactate‐*co*‐glycolate‐*co*‐d‐2‐hydroxybutyrate) was confirmed by live/dead assay using human mesenchymal stem cells.

## Introduction

Poly(lactate‐*co*‐glycolate), PLGA, is a random copolymer of lactic and glycolic acids and is approved by Food and Drug Administration and European Medicine Agency for various biomedical and therapeutic applications, such as surgical sutures, prosthetic devices, drug delivery and tissue engineering due to its biocompatibility and biodegradability with controlled degradation characteristics (Makadia and Siegel, 2011; Danhier *et al*., 2012). Currently, it is synthesized by random ring‐opening copolymerization of lactide and glycolide, the cyclic dimers of lactic and glycolic acids, respectively, using metal catalysts (Wu and Wang, 2001). Such chemical methods are rather complicated as they involve the production and purification of monomers, synthesis of pre‐polymer and removal of residual monomers and catalysts.

As a more environmentally friendly method, we recently reported production of PLGA and various d‐lactate and glycolate containing polyhydroxyalkanoates (PHAs) by metabolically engineered *Escherichia coli* strains from renewable resources (Choi *et al*., 2016). In the above study, the xylose metabolizing Dahms pathway of *Caulobacter crescentus* was established in *E. coli* by introducing the xylose dehydrogenase (XylB) and xylonolactonase (XylC) of *C. crescentus*. It resulted in production of glycolate from xylose, but also resulted in significant growth retardation; in this study, the acid names, d‐lactate and glycolate, are generally used unless they are free acids excreted into the medium (d‐lactic acid and glycolic acid).

Based on the *in silico* simulations, *E. coli* was engineered to utilize glucose and xylose simultaneously by inactivating glucose phosphotransferase system. This co‐utilizing *E. coli* strain showed recovered cell growth with enhanced glycolate production. Further metabolic engineering was performed to more efficiently produce d‐lactate and glycolate, which were then converted to d‐lactyl and glycolyl‐CoAs by evolved propionyl‐CoA transferase (Pct540) followed by polymerization to PLGA by evolved PHA synthase (PhaC1437). The engineered *E. coli* produced PLGA to 1.95 g l^−1^ with a polymer content of 36.2 wt% (Choi *et al*., 2016). It was also interesting to note that poly(d‐lactate‐*co*‐glycolate‐*co*‐d‐2‐hydroxybutyrate) [poly(d‐LA‐*co*‐GA‐*co*‐d‐2HB)] was produced when there was no manipulation of *ilvA* or supplementation of L‐isoleucine. Thus, we became interested in examining whether this terpolyester is also biocompatible like PLGA. In this study, we focused on the Dahms pathway utilizing xylose to improve the production of PLGA and poly(d‐LA‐*co*‐GA‐*co*‐d‐2HB). Effects of employing five different synthetic promoters to express XylBC_*ccs*_ of the Dahms pathway on cell growth, polymer production and monomer fractions were examined. Using the constructed engineered strains, fed‐batch cultures were performed to compare their performance with respect to cell growth and polymer production. Also, biocompatibility of poly(d‐LA‐*co*‐GA‐*co*‐d‐2HB) was examined for the first time by Live/Dead assay using human mesenchymal stem cells (hMSCs).

## Results and discussion

### Effects of XylBC_*ccs*_ expression levels on cell growth and metabolites production

**Figure 1:**
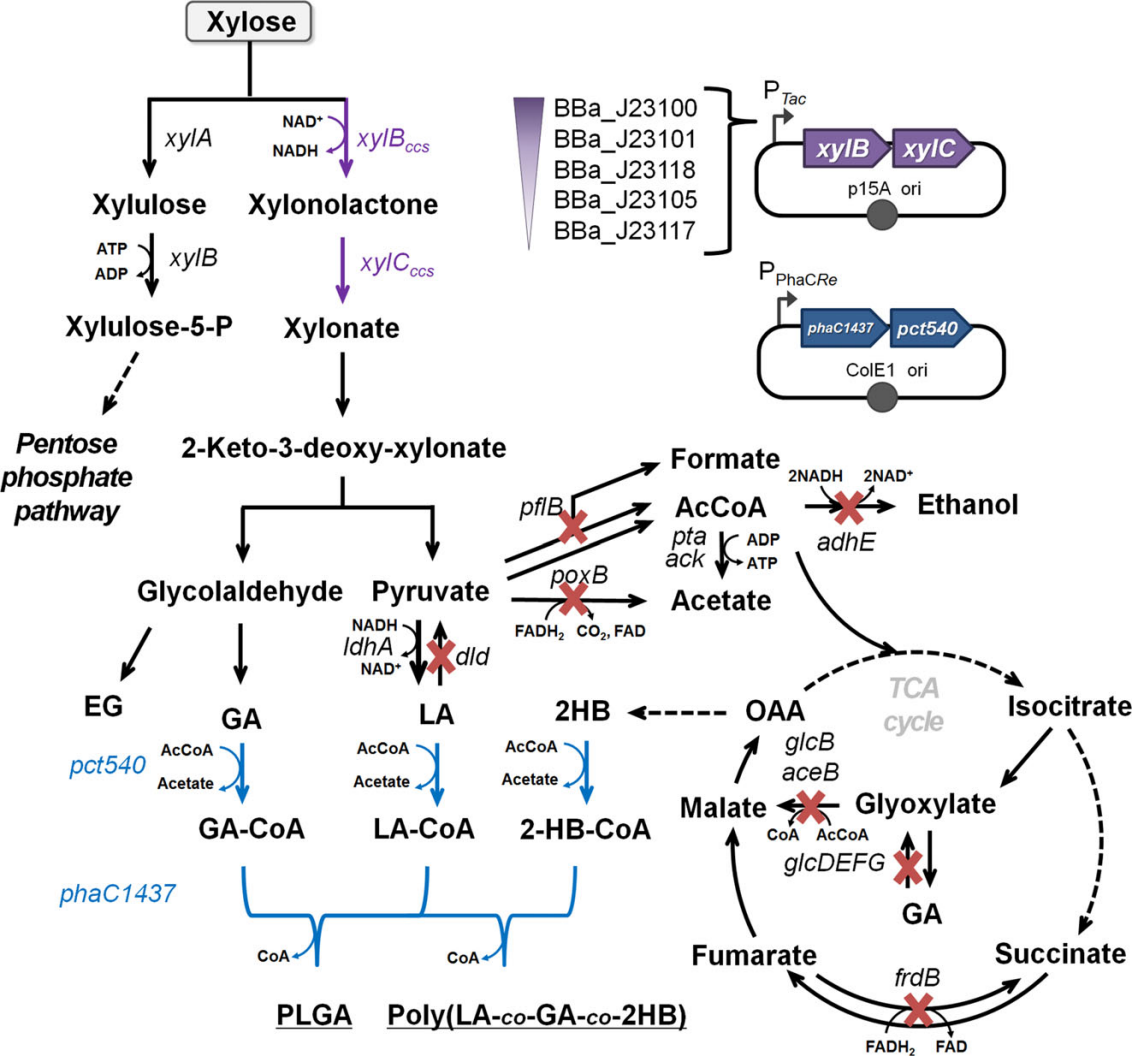
Metabolic engineering of *Escherichia coli* for the production of PLGA and its copolymers from xylose. The overall strategies for the production of PLGA and its copolymers are shown. The native pathways in *E. coli* are shown in black arrow. Coloured arrows represent heterologous pathways introduced. The inactivated metabolic pathways are indicated by red X, and strengthened metabolic pathway by replacement of native promoter is shown in bold arrow. More than one conversion steps of metabolic pathways are simplified using dotted arrows. The genes shown are as follows: *xylA*, xylose isomerase; *xylB*, xylulokinase; *xylB*_*ccs*_, xylose dehydrogenase; *xylC*_*ccs*_, xylonolactonase; *ldhA*, d‐lactate dehydrogenase; *pflB*, pyruvate formate lyase; *poxB*, pyruvate oxidase; *adhE*, acetaldehyde/alcohol dehydrogenase; *frdB*, fumarate reductase; *pta*, phosphotransacetylase; *ack*, acetate kinase; *aceB*, malate synthase A; *glcB*, malate synthase G; *glcDEFG*, glycolate oxidase; *pct540*, evolved propionyl‐CoA transferase; *phaC1437*, evolved PHA synthase. Metabolites shown are as follows: Xylulose‐5‐P, xylulose‐5‐phosphate; AcCoA, acetyl‐CoA; LA, d‐lactate; GA, glycolate; EG, ethylene glycol; 2HB, d‐2‐hydroxybutyrate; GA‐CoA, glycolyl‐CoA; LA‐CoA, d‐lactyl‐CoA; 2‐HB‐CoA, 2‐d‐hydroxybutyryl‐CoA; OAA, oxaloacetate.

**Figure 2:**
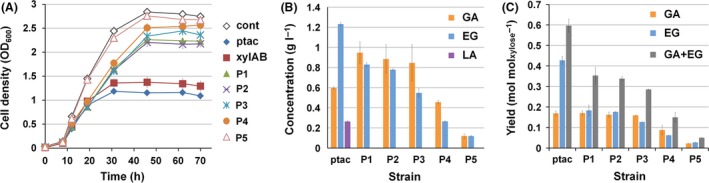
Flask cultures of *Escherichia coli* XL1‐blue expressing XylBC_*ccs*_ using different promoters. A. The time profiles of cell density (OD_600_). B. Production of glycolic acid and ethylene glycol, and (C) metabolites yield (mol mol_xylose_^−1^) at the end of cultivation (70 h). GA, EG and LA indicate glycolic acid, ethylene glycol and d‐lactic acid, respectively. The strains used were XL1‐Blue harbouring pTac15k (cont), pTacxylBC (ptac), pTacxylBC_xylAB (xylAB) and pP1xylBC‐pP5xylBC (P1–P5), respectively. Error bars represent the SD values obtained in triplicate experiments.

To produce GA *in vivo*, the heterologous Dahms pathway was established by introducing *C. crescentus* XylBC_*ccs*_ in *E. coli* XL1‐Blue strain using pTacxylBC plasmid as previously described (Choi *et al*., 2016; Table 1). When *E. coli* XL1‐Blue harbouring pTacxylBC was cultivated using xylose as a sole carbon source, 0.60 g l^−1^ of glycolic acid was produced. As before (Choi *et al*., 2016), significant cell growth retardation was observed and the final cell density (OD_600_) was 1.09, which corresponds to only 39.7% of that obtained with the control strain *E. coli* XL1‐Blue harbouring an empty vector pTac15k (Figs 1 and 2 and Table 1).

**Table 1:** All bacterial strains and plasmids used in this study

Strains and plasmids	Relevant characteristics^a^	Reference or Source
*Strains*		
XL1‐Blue	*recA1 endA1 gyrA96 thi‐1 hsdR17 supE44 relA1 lac* [F′ *proAB lacI* ^*q*^ *ZΔM15* Tn*10* (Tet^R^)]	Stratagene^b^
X15	XL1‐Blue *ΔpflB ΔfrdB ΔadhE ΔpoxB*	This study
X15l	X15 *PldhA::Ptrc*	This study
X15ld	X15 *PldhA::Ptrc Δdld*	This study
X15lda	X15 *PldhA::Ptrc Δdld Δackpta*	This study
X17ld	X15 *PldhA::Ptrc Δdld ΔaceB ΔglcDEFGB*	This study
*Plasmids*		
pPs619C1437Pct540	pBluescriptII KS(+) derivative; *R. eutropha* PHA biosynthesis operon promoter of the, *phaC1*_*Ps*6–19_ variant (*phaC1437*; E130D, S325T, S477G, Q481K), *pct*_*Cp*_ variant (*pct540*; V193A, Silent mutations: T78C, T669C, A1125G, T1158C), transcriptional terminator of the *R. eutropha* PHA biosynthesis operon; Ap^R^	Yang *et al*. (2011)
pTac15k	pACYC177 derivative; *tac* promoter; Km^R^	Laboratory stock
pMloxC	lox66‐cat‐lox71 cassette, Cm^R^ Ap^R^	Kim *et al*. (2008)
pKD46	Ap^R^, λ‐Red recombinase under arabinose inducible araBAD promoter, temperature sensitive origin	Datsenko and Wanner (2000)
pJW168	Cre recombinase under IPTG inducible lacUV5 promoter, temperature sensitive origin, Ap^R^	Palmeros *et al*. (2000)
pMtrc9	pMloxC derivative, *trc* promoter downstream of lox66‐cat‐lox71 casette, Ap^R^	Laboratory stock
pTacxylBC	pTac15k derivative; *tac* promoter*, C. crescentus xylBC* genes; Km^R^	Choi *et al*. (2016)
pTacxylBC_xylAB	pTac15k derivative; *tac* promoter*, C. crescentus xylBC* genes, *Escherichia coli* W3110 *xylAB* genes; Km^R^	This study
pP1xylBC	pTac15k derivative; BBa_J23100 promoter*, C. crescentus xylBC* genes; Km^R^	This study
pP2xylBC	pTac15k derivative; BBa_J23101 promoter*, C. crescentus xylBC* genes; Km^R^	This study
pP3xylBC	pTac15k derivative; BBa_J23118 promoter*, C. crescentus xylBC* genes; Km^R^	This study
pP4xylBC	pTac15k derivative; BBa_J23105 promoter*, C. crescentus xylBC* genes; Km^R^	This study
pP5xylBC	pTac15k derivative; BBa_J23117 promoter*, C. crescentus xylBC* genes; Km^R^	This study

a Ap, ampicillin; Km, Kanamycin; R, resistance.

b Stratagene Cloning System, La Jolla CA, USA.

To further investigate the relationship between cell growth and the Dahms pathway flux, *in silico* simulation was performed (Fig. S1). When XylBC_*ccs*_ were expressed, *E. coli* was able to catabolize xylose via two metabolic pathways: the heterologous Dahms pathway and the native *E. coli* xylose catabolic pathway via the pentose phosphate pathway (Fig. 1). Therefore, after addition of the XylBC_*ccs*_ reactions into the *E. coli* iJO1336 model, the maximum growth rate was examined by varying the relative ratio of utilizing Dahms pathway and native xylose catabolic pathway in the presence of xylose as a sole carbon source (Fig. S1). The result showed that there was an inversely proportional relationship between the Dahms pathway flux and the maximum growth rate (Fig. S1). Based on the experimental and simulation results, it was concluded that the Dahms pathway affected cell growth more negatively compared with the native *E. coli* xylose catabolic pathway.

Thus, it was attempted to strengthen the metabolic flux through the native *E. coli* xylose catabolic pathway to enhance cell growth. In *E. coli*, xylose is converted by xylose isomerase (XylA) to xylulose, which is then phosphorylated to xylulose‐5‐phosphate by xylulose kinase (XylB). Xylulose‐5‐phosphate is further metabolized via pentose phosphate pathway (Fig. 1). The *E. coli* XylA and XylB were overexpressed together with XylBC_*ccs*_ using plasmid pTacxylBC_xylAB (Table 1). However, this resulted in only 1.19‐fold increase in final cell density, which was 47% of control strain *E. coli* XL1‐Blue harbouring an empty vector pTac15k (Fig. 2A).

As enhancing the native xylose pathway flux was not effective, reducing the Dahms pathway flux itself was chosen as an alternative strategy. However, too much reduction in the Dahms pathway flux can potentially reduce GA production and consequently decreased PLGA production. Thus, the optimal metabolic flux to maximize the PLGA production was examined by adjusting the Dahms pathway flux. To achieve this, we constructed five plasmids pP1xylBC, pP2xylBC, pP3xylBC, pP4xylBC and pP5xylBC that allow expression of XylBC_*ccs*_ under five different synthetic Anderson promoters, BBa_J23100, BBa_J23101, BBa_J23118, BBa_J23105 and BBa_J23117 (Table 1), respectively, instead of the *tac* promoter originally employed. These constitutive promoters, BBa_J23100 (P1 hereafter for convenience), BBa_J23101 (P2), BBa_J23118 (P3), BBa_J23105 (P4), and BBa_J23117 (P5), have relative promoter strengths of 1, 0.7, 0.56, 0.24 and 0.06, respectively (http://parts.igem.org/).

When *E. coli* XL1‐Blue transformed with each XylBC_*ccs*_ expression plasmid was cultivated, the OD_600_ profile showed that the cell concentration was increased as the promoter strength decreased (Fig. 2A). Even when XylBC_*ccs*_ were expressed under P1 promoter, the strongest one among the five synthetic promoters, cell density was restored to 81.2% of the control strain (*E. coli* XL1‐Blue harbouring an empty vector pTac15k). When the weakest P5 promoter was employed, cell density reached was 97.4% of that obtained with the control strain (Fig. 2A).

As shown in Fig. 2B, production of GA and ethylene glycol, which are both Dahms pathway metabolites, was found to be highly dependent on the strength of the promoter employed, and showed decreasing tendency with weaker promoter (except for the *tac* promoter). To compare the relative extent of Dahms pathway utilization in each strain, the molar yields GA and ethylene glycol on xylose were calculated (Fig. 2C). The sum of the yields of GA and ethylene glycol clearly showed a decreasing tendency with decrease in promoter strength (Fig. 2C). These results confirm that the Dahms pathway flux could be effectively modulated by controlling expression levels of XylBC_*ccs*_ using the synthetic promoters having various strengths. Also, use of the top three strong promoters (P1, P2 and P3) resulted in glycolic acid production to 0.96, 0.88 and 0.85 g l^−1^, which are higher than that (0.60 g l^−1^) obtained using the *tac* promoter. Although the relative flux towards Dahms pathway was reduced by employing a weaker promoter, enhanced cell growth led to higher level of GA production. These results demonstrate that modulating the Dahms pathway flux is effective for improving glycolic acid production.

### Metabolic engineering for d‐lactate production

*Escherichia coli* XL1‐Blue expressing XylBC_*ccs*_ using five different synthetic promoters produced 0.11–0.95 g l^−1^ of glycolic acid and negligible amount of d‐lactic acid at the end of cultivation (70 h) (Fig. 2B). Because the reported PLGA producers (Choi *et al*., 2016) were developed based on a *ptsG‐*deleted *E. coli* strain, we newly constructed *E. coli* strains to efficiently produce D‐lactate using the *E. coli* XL1‐Blue strain as a base strain, while employing the effective engineering targets found from the previous study. *E. coli* was engineered by knocking out the *poxB* (encoding pyruvate oxidase), *pflB* (encoding pyruvate formate lyase), *frdB* (encoding fumarate reductase) and *adhE* (encoding acetaldehyde/alcohol dehydrogenase) genes in the chromosome of XL1‐Blue (Fig. 1). Then, the native promoter of the *ldhA* gene (encoding d‐lactate dehydrogenase) was replaced with the strong *trc* promoter and the *dld* (encoding d‐lactate dehydrogenase) gene was deleted to construct X15ld strain (Fig. 1 and Table 1).

**Figure 3:**
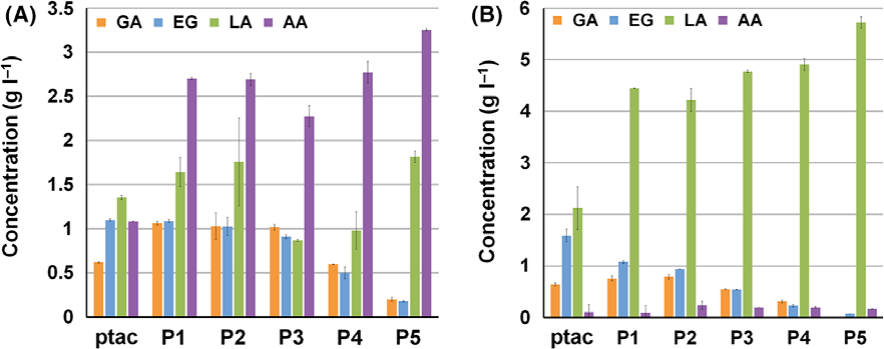
Production of glycolic and d‐lactic acids and major by‐products. Flask cultures of (A) X15ld strain and (B) X15lda strain harbouring different XylBC_*ccs*_ expression vectors. GA, EG, LA and AA indicate glycolic acid, ethylene glycol, d‐lactic acid and acetic acid, respectively. Error bars represent the SD values obtained in triplicate experiments.

Into the X15ld strain, the above plasmids expressing XylBC_*ccs*_ under different promoters (*tac* or P1‐P5) were introduced. Flask cultures of these engineered X15ld strains produced up to 1.81 g l^−1^ of d‐lactic acid (Fig. 3A). Use of the weakest P5 promoter resulted in the highest d‐lactic acid titre of 1.81 g l^−1^, while the relatively mid‐strength P3 and P4 promoters resulted in lower d‐lactic acid production of 0.87 and 0.98 g l^−1^, respectively. As cell growth was enhanced, acetic acid accumulation was also significantly increased (Fig. 3A). To reduce acetic acid production, the *ack‐pta* genes (encoding acetate kinase and phosphotransacetylase) were deleted from X15ld strain to make X15lda strain. Removal of *ack‐pta* genes effectively eliminated acetic acid formation and significantly increased d‐lactic acid production up to 5.72 g l^−1^, which is 3.1‐fold higher than that obtained with X15ld strain employing P5 promoter (Fig. 3B).

### Biosynthesis of PLGA and poly(d‐LA‐co‐GA‐co‐d‐2HB)

**Figure 4:**
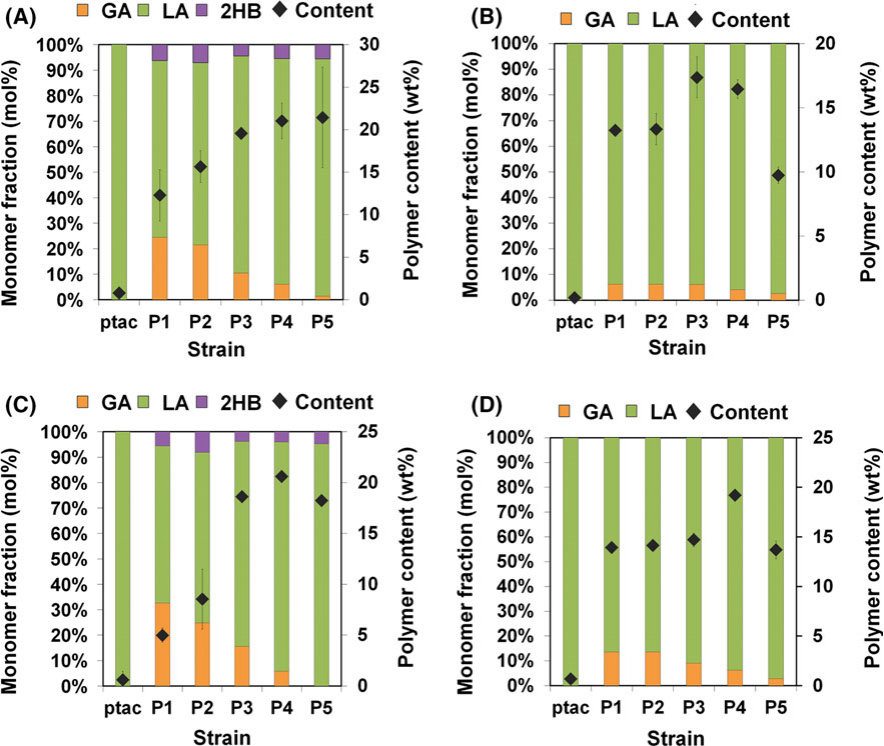
Polymer contents and compositions. Polymers produced by X15ld harbouring different XylBC_*ccs*_ expression vectors and pPs619C1437Pct540 in medium (A) alone or (B) with 2 mM l‐isoleucine supplementation. Polymers produced by X17ld harbouring different XylBC_*ccs*_ expression vectors and pPs619C1437Pct540 in medium (C) alone or (D) with 2 mM l‐isoleucine supplementation. GA, LA and 2HB indicate glycolic acid, D‐lactic acid and D‐2‐hydroxybutyric acid, respectively. The polymer contents are represented by diamonds. Error bars represent the SD values obtained in triplicate experiments.

For the biosynthesis of PLGA and poly(d‐LA‐*co*‐GA‐*co*‐d‐2HB), engineered *C. propionicum* propionyl‐CoA transferase (Pct540) and engineered *Pseudomonas* sp. MBEL 6–19 PHA synthase (PhaC1437) were chosen as these two enzymes allowed successful production of PLGA and poly(d‐LA‐*co*‐GA‐*co*‐d‐3‐hydroxybutyrate) in our previous study (Choi *et al*., 2016); plasmid pPs619C1437Pct540 was employed for expressing Pct540 and PhaC1437. The X15ld and X15lda strains harbouring pPs619C1437Pct540 and different XylBC_*ccs*_ expression vectors were cultured. Flask cultures of these engineered strains produced PLA, PLGA and poly(d‐LA‐*co*‐GA‐*co*‐d‐2HB) having various monomer compositions (Fig. 4 and Fig. S2). When XylBC_*ccs*_ was expressed under the *tac* promoter in X15ld, only small amount of PLA was produced (Fig. 4A). The X15ld strains expressing XylBC_*ccs*_ under P1‐P5 promoters produced poly(d‐LA‐*co*‐GA‐*co*‐d‐2HB)s. As the promoter strength decreased from P1 to P5, the GA mole fractions were decreased from 24.4 mol% to 1.4 mol%, while the polymer contents were increased from 12.3 wt% to 21.4 wt% (Fig. 4A). The X15lda produced polymers having higher d‐LA fractions due to the increased d‐LA flux as described above (Fig. S2). However, the polymer contents obtained were significantly lower than those (~21.4 wt%) obtained with X15ld; the highest polymer content obtained was 7.3 wt% with X15lda strain harbouring pP5xylBC and pPs619C1437Pct540. The GA mole fractions were also decreased except for the *tac* promoter case in which the increase of d‐LA production resulted in enhanced PLGA production. The GA mole fractions were only 5.1 and 2.1 mol% when P1 and P2 promoters were used, respectively. When P3, P4 and P5 promoters were employed, only poly(d‐LA‐*co*‐d‐2HB) was produced without GA incorporation (Fig. S2). As X15lda strain was not effective for PLGA production, X15ld strain was used for further study.

The produced polymers contained d‐2HB up to 7.0 mol% (Fig. 4A). This d‐2HB incorporation has been previously reported to be originated from 2‐ketobutyrate, which is an intermediate of the endogenous l‐isoleucine biosynthetic pathway in *E. coli* (Choi *et al*., 2016; Yang *et al*., 2016). To prevent d‐2HB incorporation into polymers, threonine dehydratase (IlvA), an enzyme responsible for 2‐ketobutyrate formation, was inactivated by feeding 2 mM l‐isoleucine in culture medium as IlvA is allosterically inhibited by l‐isoleucine (Umbarger, 1956). l‐Isoleucine supplementation effectively eliminated d‐2HB incorporation and resulted in production of PLGA (Fig. 4B). However, most of the strains produced polymers with reduced polymer content and GA mole fraction (~ 6.2 mol%) (Fig. 4B). Although previously verified, we analysed an example polymer produced by X15ld harbouring pPs619C1437Pct540 and pP4xylBC, poly(88.5 mol% d‐LA‐*co*‐6.1 mol% GA‐*co*‐5.4 mol% d‐2HB), by 1D (^1^H and ^13^C) NMR spectroscopy (Fig. S3). The results clearly confirmed again that poly(d‐LA‐*co*‐GA‐*co*‐d‐2HB) was produced in the engineered *E. coli*.

### Enhanced PLGA production by fed‐batch cultivation

**Figure 5:**
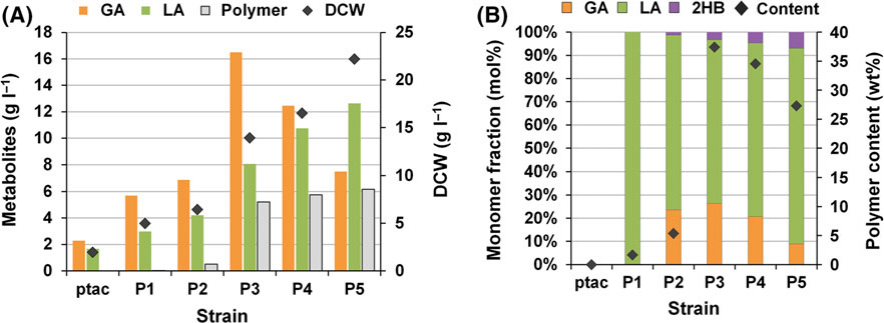
Fed‐batch cultures of X15ld expressing XylBC_*ccs*_, PhaC1437 and Pct540. (A). Concentrations of dry cell weight (DCW) and metabolites (d‐lactic and glycolic acids and polymer), and (B) polymer contents and monomer compositions at the end of fed‐batch cultures. The strains used were X15ld harbouring pPs619C1437Pct540 and different XylBC_*ccs*_ expression vectors: pTacxylBC (ptac) and pP1xylBC‐pP5xylBC (P1‐P5). GA, LA and 2HB indicate glycolic acid, d‐lactic acid and d‐2‐hydroxybutyric acid, respectively.

To examine whether PLGA production can be further enhanced and the polymer compositions can be varied, fed‐batch cultures of the X15ld harbouring pPs619C1437Pct540 and different XylBC_*ccs*_ expression vectors were performed. Cell concentrations (dry cell weight, DCW, g l^−1^) reached were 1.95, 4.98, 6.43, 13.91, 16.52 and 22.2 g DCW l^−1^ when the *tac*, P1, P2, P3, P4 and P5 promoters were employed, respectively (Fig. 5A and Fig. S4). As can be seen from these fed‐batch fermentation results, higher cell concentration could be reached when weaker promoter was employed for the expression of XylBC_*ccs*_; the strain expressing XylBC_*ccs*_ under the weakest P5 promoter showed 11.4‐fold higher cell concentration than that obtained with the strain expressing XylBC_*ccs*_ under the *tac* promoter. These results again show the negative relationship between cell growth and Dahms pathway flux modulated through adjusting the promoter strength.

Among the six engineered strains examined, X15ld harbouring pP3xylBC and pPs619C1437Pct540 resulted in the highest glycolic acid titre of 16.49 g l^−1^ (Fig. 5A and Fig. S4). When the promoters *tac*, P1, P2, P3, P4 and P5 were employed, glycolic acid titre obtained was 2.28, 5.69, 6.88 16.49, 12.46 and 7.51 g l^−1^, respectively. Glycolic acid production increased as the promoter strength decreased up to P3 promoter and then started to decrease with further reduced promoter strength. So, as observed in flask cultures, enhanced cell growth by employing weak promoters positively affected GA titre. However, use of too weak promoter resulted in overall lower GA titre due to too much decreased Dahms pathway flux. Thus, it is important to optimize the key pathway flux (e.g. Dahms pathway) affecting cell growth for enhanced GA production. Ethylene glycol production also showed similar patterns (Fig. 5A and Fig. S4). Ethylene glycol titre was highest with P1 promoter (11.69 g l^−1^) and became lower as the promoter strength was further decreased. In the case of d‐lactic and acetic acids, the production titres were inversely proportional to the promoter strength (Fig. 5A and Fig. S4). As promoter strength decreased from *tac* to P5, production of d‐lactic acid and acetic acid increased from 1.66 to 12.64 g l^−1^ and 1.70 to 10.23 g l^−1^, respectively. (Fig. 5A and Fig. S4).

When the *tac* and P1 promoters were employed for XylBC_*ccs*_ expression, only negligible amount of polymer was produced due to insufficient d‐LA and GA production (Fig. 5A and Fig. S4). Use of P2 promoter resulted in production of only 5.3 wt% of poly(75.2 mol% d‐LA‐*co*‐23.5 mol% GA‐*co*‐1.3 mol% d‐2HB) at the end of fed‐batch culture (Fig. 5). When P3 promoter was used, poly(70.5 mol% d‐LA‐*co*‐26.3 mol% GA‐*co*‐3.2 mol% d‐2HB) was produced to the highest polymer content of 37.4 wt%, which is significantly higher than that (19.6 wt%) obtained by flask culture (Fig. 5B). Also, the highest GA mole fraction of 26.3 mol% could be obtained. When the weakest P5 promoter was used, poly(84.2 mol% d‐LA‐*co*‐8.8 mol% GA‐*co*‐7.0 mol% d‐2HB) was produced to the polymer content of 27.3 wt%. Although the polymer content was lower than those obtained with the strains employing P3 and P4 promoters, the higher cell concentration led to the highest polymer titre of 6.1 g l^−1^ (Fig. 5). The polymer mole fractions were not much changed during the fed‐batch culture (Fig. S4).

### Construction of X17ld strain to further increase the glycolate fraction

As the monomer fraction of PLGA is an important characteristic to modulate the polymer degradation rate for various applications, further metabolic engineering was performed to enhance GA production. Because GA can be converted into glyoxylate by the native GA oxidase (GlcDEFG), the corresponding *glcDEFG* genes were deleted in the chromosome of X15ld. Also, the *glcB* and *aceB* genes encoding malate synthases were deleted to concentrate glyoxylate because glyoxylate can be converted into GA by endogenous glyoxylate reductase (YcdW); this strain was named X17ld (Fig. 1). Engineered X17ld strains harbouring pPs619C1437Pct540 and different XylBC_*ccs*_ expression vectors were constructed and cultured in flasks to compare strain performances. The X17ld strains expressing XylBC_*ccs*_ under P1, P2 and P3 promoters produced poly(d‐LA‐*co*‐GA‐*co*‐d‐2HB) with higher GA mole fractions compared to those obtained with the similarly engineered X15ld strains (Fig. 4A and C). The GA mole fraction decreased with decreasing promoter strength except for the *tac* promoter. When P1 promoter was employed, polymer having 32.7 mol% GA was produced to the polymer content of 5.0 wt% (Fig. 4C). When 2 mM of l‐isoleucine was supplemented into the culture medium, the engineered X17ld strains produced PLGA containing up to 13.6 mol% of GA (for P1 promoter) that were also higher than those (up to 6.2 mol% of GA) obtained by employing similarly engineered X15ld strains (Fig. 4B and D).

**Figure 6:**
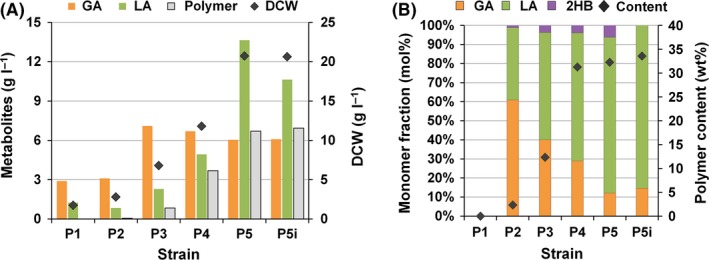
Fed‐batch cultures of X17ld expressing XylBC_*ccs*_, PhaC1437 and Pct540. (A). Concentrations of dry cell weight (DCW) and metabolites (d‐lactic and glycolic acids and polymer), and (B) polymer contents and monomer compositions at the end of fed‐batch cultures. The strains used were X17ld harbouring pPs619C1437Pct540 and different XylBC_*ccs*_ expression vectors: pP1xylBC‐pP5xylBC (P1–P5). P5i indicates that X17ld strain harbouring pPs619C1437Pct540 and pP5xylBC was cultured in l‐isoleucine‐supplemented medium. GA, LA and 2HB indicate glycolic acid, d‐lactic acid and d‐2‐hydroxybutyric acid, respectively.

Next, fed‐batch cultures of these engineered X17ld strains were performed. As the engineered strain expressing XylBC_*ccs*_ under *tac* promoter (the X17ld harbouring pTacxylBC and pPs619C1437Pct540) could not grow well, the results of this fed‐batch culture are not included. As shown in Fig. 6A and Fig. S5, the engineered X17ld strains also showed similar tendencies in cell concentration and metabolites production with those observed with similarly engineered X15ld strains. As promoter strength decreased, the concentrations of cell, d‐lactic acid and acetic acid were increased. The highest glycolic acid and ethylene glycol concentrations were 8.81 and 6.7 g l^−1^, when the X17ld employing P3 promoter was used (Fig. 6A and Fig. S5).

In the fed‐batch cultures, the GA mole fraction of produced polymers could be further increased (Fig. 6B). When P2 and P3 promoters used, GA mole fractions were 60.9 and 40.1 mol%, respectively (Fig. 6B). Similarly to the results of fed‐batch cultures of engineered X15ld strains, the highest polymer titre (6.70 g l^−1^) was obtained when the weakest P5 promoter was employed (Fig. 6A). The engineered X17ld strain employing P5 promoter produced poly(81.7 mol% d‐LA‐*co*‐12.1 mol% GA‐*co*‐6.2 mol% 2HB) to the polymer content of 32.3 wt% (Fig. 6B). To produce PLGA without incorporation of d‐2HB, l‐isoleucine was supplemented into the culture medium during fed‐batch cultures of X17ld harbouring pPs619C1437Pct540 and pP5xylBC. As a result, 6.93 g l^−1^ of poly(85.4 mol% d‐LA‐*co*‐14.6 mol% GA) could be successfully produced to a polymer content of 33.4 wt% (Fig. 6 and Fig. S5).

### Molecular weights, thermal properties and biocompatibility of poly(d‐LA‐co‐GA‐co‐d‐2HB)

As the biocompatibility of poly(d‐LA‐*co*‐GA‐*co*‐d‐2HB) is not known, we investigated it with two representative polymers, poly(70.4 mol% d‐LA‐*co*‐26.4 mol% GA‐*co*‐3.2 mol% d‐2HB) and poly(84.2 mol% d‐LA‐*co*‐8.8 mol% GA‐*co*‐7.0 mol% d‐2HB) produced by fed‐batch cultures. Before doing so, these polymers were characterized with respect to molecular weights by gel permeation chromatography (GPC) and thermal properties by differential scanning calorimetry (DSC). Previously, several studies also reported for the synthesis of 2HB containing polymers such as homo poly(d‐2HB) and copolymers including poly(d‐LA‐*co*‐d‐2HB) and characterization of molecular weight, biodegradation, thermal and mechanical properties (Park *et al*., 2012, 2013; Matsumoto *et al*., 2013; Chae *et al*., 2016).

The number average (M_n_) and weight average (M_w_) molecular weights of 26.4 mol% GA containing polymer were 10,128 and 13,304 Da, respectively, with the polydispersity index of 1.314 (Table 2). The 8.8 mol% GA containing polymer had molecular weights of 16,612 (M_n_) and 26,308 (M_w_) Da with the polydispersity index of 1.584. These molecular weights are similar to those of PLGAs produced by engineered *E. coli* strains we previously reported (Choi *et al*., 2016). Also, they are comparable to those of PLGAs suitable for use in making microsphere for drug delivery carrier and scaffolds for tissue engineering (Makino *et al*., 2004; Campos *et al*., 2014; Xie *et al*., 2015). The thermal properties of the two polymers were measured by DSC analysis (Table 2). They exhibited glass transition temperature at 45.0 and 49.9 °C, respectively, and no melting temperatures, indicating that the produced polymers are amorphous. These properties are also similar to those of chemically synthesized PLGA (Park, 1995).

**Table 2:** Polymer characteristics of two sample poly(d‐LA‐*co*‐GA‐*co*‐d‐2HB) produced by engineered *Escherichia coli*

Monomer fraction^a^ (mol%)			Molecular weight^b^			Thermal properties^c^		
LA	GA	2HB	M_n_ (Da)	M_w_ (Da)	M_w_ M_n_^−1^	T_g_	T_m_	ΔC_p_
70.4	26.4	3.2	10,128	13,304	1.314	45.0	–	0.773
84.2	8.8	7.0	16,612	26,308	1.584	49.9	–	0.638

a LA, GA and 2HB indicate d‐lactate, glycolate and d‐2‐hydroxybutyrate, respectively.

b M_n_, number average molecular weight; M_w_, weight average molecular weight; M_w_ M_n_^−1^, polydispersity index.

c T_g_, glass transition temperature (°C); T_m_, melting temperature (°C); ΔC_p_, heat capacity (J g^−1^ K^−1^).

**Figure 7:**
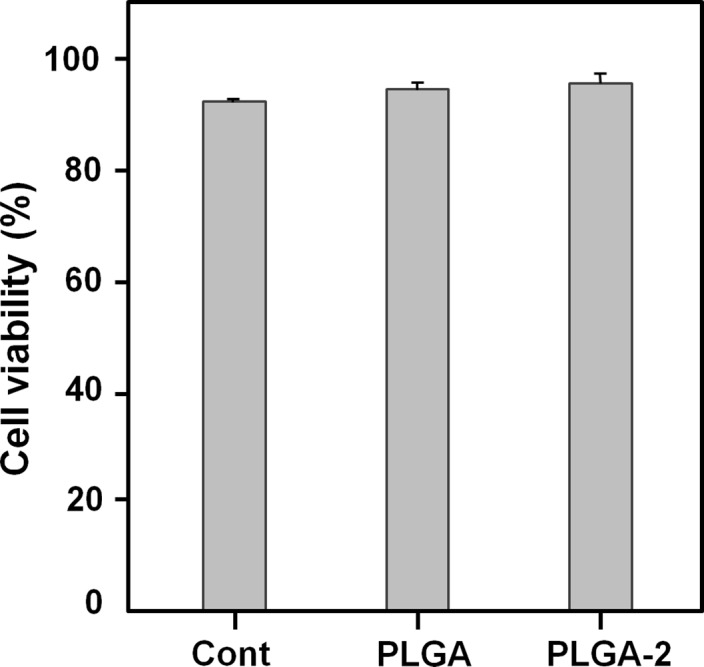
Live/dead assay. Percentage of live human mesenchymal stem cells (hMSCs) on each substrate: cover glass (Cont), PLGA‐coated glass (PLGA) or poly(d‐LA‐*co*‐GA‐*co*‐d‐2HB)‐coated glass (PLGA‐2). Error bars represent the SD values obtained in triplicate experiments.

Next, we examined the biocompatibility of one of these d‐2HB containing PLGA polymers, poly(84.2 mol% d‐LA‐*co*‐8.8 mol% GA‐*co*‐7 mol% d‐2HB). To evaluate the polymer cytotoxicity, the live/dead assay of human mesenchymal stem cell (hMSC) was performed. The hMSCs were seeded on cover glass (spin coated with each polymer) and cultivated for 4 days. The poly(84.2 mol% d‐LA‐*co*‐8.8 mol% GA‐*co*‐7 mol% d‐2HB) exhibited high cell viability similar to that of commercial PLGA (Fig. 7 and Fig. S6). This result demonstrated that poly(d‐LA‐*co*‐GA‐*co*‐d‐2HB) containing d‐2HB as an additional monomer is biocompatible.

## Conclusion

We recently reported one‐step direct fermentative production of PLGA, poly(d‐LA‐*co*‐GA‐*co*‐d‐2HB), and other PLGA copolymers by metabolically engineered *E. coli* strains (Choi *et al*., 2016). As the introduction of heterologous Dahms pathway producing GA negatively affected cell growth, the metabolic flux towards Dahms pathway was modulated using five synthetic promoters of different strengths. Through further metabolic engineering described above, PLGA and poly(d‐LA‐*co*‐GA‐*co*‐d‐2HB) with various monomer compositions could be more efficiently produced. Also, the GA mole fraction in poly(d‐LA‐*co*‐GA‐*co*‐d‐2HB) could be modulated between 8.8 and 60.9 mol% of GA. The polymer titre could also be increased up to 6.93 g l^−1^ in the case of poly(85.4 mol% d‐LA‐*co*‐14.6 mol% GA). As poly(d‐LA‐*co*‐GA‐*co*‐d‐2HB) has been less characterized, their molecular weights, thermal properties and biocompatibility were examined. Poly(d‐LA‐*co*‐GA‐*co*‐d‐2HB) was found to be biocompatible and thus can be similarly used for medical applications like PLGA.

This microbial production system can be further improved with respect to polymer titre, yield and productivity by adapting various systems metabolic engineering strategies (Lee *et al*., 2011, 2012; Choi *et al*., 2016; Lee and Kim, 2015; Song and Lee, 2015). One of the bottlenecks is less optimal PHA synthase activity and specificity. As the crystal structure of PHA synthase from *Ralstonia eutropha* has been recently resolved (Wittenborn *et al*., 2016; Kim *et al*., 2017a,b), much better and rational engineering of PHA synthase based on the structural information will improve the bio‐based production of PLGA and various polymers.

## Experimental procedures

### Bacterial strains and plasmids

All bacterial strains, plasmids and primers used in this study are listed in Table 1 and Table S1. *E. coli* XL1‐Blue (Stratagene Cloning Systems, La Jolla, CA, USA) was used for general gene cloning studies. *E. coli* XL1‐Blue and its mutants were used as host strains for the synthesis of PLGA and PLGA copolymers. All DNA manipulations were performed following standard procedures (Sambrook and Russell, 2001). PCR was performed with the C1000 Thermal Cycler (Bio‐Rad, Hercules, CA, USA).

Plasmid pPs619C1437Pct540, which expresses the *Pseudomonas* sp. MBEL 6–19 PHA synthase containing quadruple mutations of E130D, S325T, S477G, and Q481K (PhaC1437) and the *Clostridium propionicum* propionyl‐CoA transferase mutant containing V193A and four silent nucleotide mutations of T78C, T669C, A1125G, and T1158C (Pct540) under the *Ralstonia eutropha* PHA biosynthesis operon promoter, has been previously described (Table 1; Yang *et al*., 2011). Plasmid pTacxylBC expressing *Caulobacter crescentus* XylBC_*ccs*_ (xylose dehydrogenase and xylonolactonase) has also been reported (Table 1; Choi *et al*., 2016).

To construct pTacxylBC_xylAB plasmid, *E. coli xylAB* genes encoding xylose isomerase and xylulose kinase were amplified from the chromosomal DNA of *E. coli* XL1‐Blue by PCR using the primers xylAB_F and xylAB_R (Table S1). For expression of the XylAB under *tac* promoter, the *tac* promoter region was amplified from the pTac15k using the primers tac_xylAB_F and tac_xylAB_R. The *xylAB*,* tac* and linear DNA of pTacxylBC digested by PstI and SphI were assembled into pTacxylBC_xylAB (Table 1 and Table S1).

To express XylBC_*ccs*_ with different strength, the five Anderson promoters (BBa_J23100, BBa_J23101, BBa_J23118, BBa_J23105 and BBa_J23117) were used (http://parts.igem.org). To construct pP1xylBC plasmid, pTacxylBC plasmid was amplified by inverse PCR using the primer 100_F and 100_R to replace the original *tac* promoter with the BBa_J23100 promoter. The construction of pP2xylBC‐pP5xylBC plasmids was performed in the same manner (Table 1 and Table S1).

### Deletion of chromosomal genes and promoter replacement

Deletion of *E. coli* chromosomal genes was achieved using one‐step inactivation method as previously described (Datsenko and Wanner, 2000). The primers and detailed methods are described in previous paper (Choi *et al*., 2016). Briefly, the linear DNA fragment having homologous region with the target gene was prepared by PCR using pMloxC (Kim *et al*., 2008). The PCR fragments were introduced by electroporation into the recombinant *E. coli* harbouring pKD46 to express λ recombinase (Datsenko and Wanner, 2000). The mutants in which double homologous recombination occurred were selected, and then, the chloramphenicol resistance gene was eliminated by introducing pJW168 encoding the *cre* recombinase in presence of 1 mM isopropyl β‐D‐1‐thiogalactopyranoside (IPTG; Sigma‐Aldrich, St. Louis, MO, USA; Palmeros *et al*., 2000). For the replacements of chromosomal *ldhA* promoter, plasmid pMtrc9 having *trc* promoter at the downstream of lox66‐cat‐lox71 cassette was used in place of pMloxC.

### Cultivation and metabolites analysis

*Escherichia coli* was routinely cultured at 37 °C in Luria‐Bertani (LB) medium containing 10 g l^−1^ tryptone, 5 g l^−1^ yeast extract and 10 g l^−1^ NaCl. For the production, recombinant *E. coli* strains were cultured in MR medium supplemented with 100 mM 3‐(N‐morpholino)propansulfonic acid (MOPS) in 30 °C in a rotary shaker at 250 rpm. The MR medium (pH 7.0) contains (per litre) 6.67 g KH_2_PO_4_, 4 g (NH_4_)_2_HPO_4_, 0.8 g MgSO_4_·7H_2_O, 0.8 g citric acid and 5 ml trace metal solution. The trace metal solution contains (per litre of 0.5 M HCl) 10 g FeSO_4_·7H_2_O, 2 g CaCl_2_, 2.2 g ZnSO_4_·7H_2_O, 0.5 g MnSO_4_·4H_2_O, 1 g CuSO_4_·5H_2_O, 0.1 g (NH_4_)_6_Mo_7_O_24_·4H_2_O and 0.02 g Na_2_B_4_O_7_·10H_2_O. Xylose and MgSO_4_·7H_2_O were sterilized separately. Seed cultures were prepared in 25‐ml test tubes containing 5‐ml LB medium at 30 °C overnight in a rotary shaker at 250 rpm. One ml of overnight culture was used to inoculate 250‐ml flask containing 100 ml of 100 mM MOPS added MR medium containing 10 mg l^−1^ of thiamine supplemented with 20 g l^−1^ of xylose. Ampicillin (Ap, 50 μg ml^−1^) and kanamycin (Km, 30 μg ml^−1^) were added to the medium depending on the resistance marker of the employed plasmid. One mM of IPTG (Sigma Aldrich) was added at the beginning of the flask cultivation. To inactivate IlvA, 2 mM of l‐isoleucine was added to the culture medium.

Fed‐batch cultures carried out in a 6.6L Bioreactor (Bioflo 3000, New Brunswick Scientific, Edison, NJ, USA) containing 100 mM MOPS added MR medium supplemented with 20 g l^−1^ of xylose, and 10 mg l^−1^ of thiamine. The dissolved oxygen concentration (DOC) was maintained above 10% of air saturation by changing the agitation speed. The pH was controlled at 7.0 by ammonia solution. The following feeding solution was used: 700 g l^−1^ xylose plus 0.8 g MgSO_4_·7H_2_O and 10 mg thiamine. The feeding solution was manually fed into the fermentor when the xylose concentration dropped to below 5 g l^−1^ to increase it to about 20–25 g l^−1^. For l‐isoleucine‐supplemented culture, the l‐isoleucine was manually fed into the fermentor when the l‐isoleucine concentration dropped to below 1 mM to increase it to about 2 mM.

The carbon sources (xylose) and metabolites (acetate, glycolate, d‐lactate, ethylene glycol, formate, succinate) were measured by high‐performance liquid chromatography (HPLC) (1515 isocratic HPLC pump; Waters, Milford, MA, USA) equipped with refractive index detector (2414; Waters). A MetaCarb 87H column (Agilent, Palo Alto, CA, USA) was eluted isocratically with 0.01 N H_2_SO_4_ at 25 °C at flow rate of 0.5 ml min^−1^. The amino acid (l‐isoleucine) was measured as previously described (Shin *et al*., 2016). For the detection of amino compounds, the supernatant of the culture was reacted with *o*‐phthalaldehyde and the derivatized amino acids were analysed by Agilent 1100 HPLC (Agilent). Cell growth was monitored by measuring the absorbance at 600 nm (OD_600_) using an Ultrospec 3000 spectrophotometer (Amersham Biosciences, Uppsala, Sweden).

### Polymer analysis

To analyse the contents and monomer compositions of polymers produced by engineered *E. coli*, the samples were prepared as previously described and were analysed by gas chromatography (GC; Braunegg *et al*., 1978; Choi *et al*., 2016)_._ The cells were washed with distilled water and then lyophilized. The polymers in lyophilized cells were converted into corresponding hydroxymethyl esters by acid‐catalysed methanolysis. The resulting methyl esters were measured by GC (Agilent 6890N; Agilent) equipped with Agilent 7683 automatic injector, flame ionization detector and a fused silica capillary column (ATTM‐Wax, 30 m, ID 0.53 mm, film thickness 1.20 m; Alltech, Deerfield, IL, USA). Polymer content is defined as the weight percentage of polymer concentration to dry cell concentration (wt% of dry cell weight).

Polymers were extracted from the cells by the chloroform extraction (Choi and Lee, 1999). The harvested cells were washed with distilled water and lyophilized. The lyophilized cells were located in chloroform refluxed Soxhlet apparatus (Corning, Lowell, MA, USA) overnight. The chloroform solution was concentrated and then precipitated by addition of ice cold methanol. The structure, molecular weight and thermal properties of the polymers were determined by nuclear magnetic resonance (NMR) spectroscopy, gel permeation chromatography (GPC) and differential scanning calorimetry (DSC), respectively, as previously described (Jung and Lee, 2011).

### Biocompatibility test

The polymers were dissolved in chloroform at 2 w v^−1^%. The solution of each polymer was dropped on 18‐mm cover glass and spin coated at 1200 *g* for 1 min. The spin‐coated cover glass was kept at room temperature for overnight to allow evaporation of chloroform. The cover glass and spin‐coated cover glass were washed with 1X Dulbecco's phosphate‐buffered saline for 3 times and cured by ultraviolet light for 30 min for sterilization. The human mesenchymal stem cells (Lonza, Muenchensteinerstrasse 38, CH‐4002 Basel, Switzerland) were seeded onto the sterilized cover glasses and polymer films at a seeding density of 2.5 × 10^4^ cells cm^−2^ and cultured for 4 days with Minimum Essential Media alpha (α‐MEM, Gibco, Grand Island, NY, USA) supplemented with 17% foetal bovine serum (FBS, Welgene, Gyeongsan‐si, Gyeongsangbuk‐do, Republic of Korea), 100 units ml^−1^ penicillin (Gibco) and 100 μg ml^−1^ streptomycin (Gibco) in 5% CO_2_ and 37 °C incubator. The medium is exchanged every 2 days.

A live/dead assay is performed using LIVE/DEAD^®^ Viability/Cytotoxicity Assay Kit for mammalian cells (Invitrogen, Carlsbad, CA, USA). The kit reagents, Calcein AM and ethidium homodimer‐1 (EthD‐1), are diluted in 1X Dulbecco's phosphate‐buffered saline (DPBS) according to the kit instructions. The cells cultured on cover glasses and PLGA films were gently washed with 1X DPBS for 3 times, and 1 ml of diluted Calcein AM and EthD‐1 solution was added to the cover glasses and PLGA films for 45 min at room temperature. The live cells were stained with Calcein AM, and dead cells were stained with EthD‐1. After staining, cells were gently washed with 1X DPBS for 3 times again and imaged with a fluorescence microscope (Ti‐U; Nikon, Minato‐ku, Tokyo, Japan). The percentage of live and dead cells was counted with imagej software (https://imagej.nih.gov/ij/) with three independent experiments.

## Conflict of interest

The authors declare that they have conflict of interest as the related technologies are patented and under commercialization discussion.

## Supporting information


**Table S1.** Primers used in this study.
**Fig. S1.** *In silico* genome‐scale analysis of the cell maximum growth rate changes caused by introducing Dahms pathway.
**Fig. S2.** Polymer contents and compositions of polymers produced by X15lda strain harboring pPs619C1437Pct540 and different XylBC_*ccs*_ expression vectors.
**Fig. S3.** NMR analysis. (A) ^1^H NMR spectrum and (B) ^13^C NMR spectrum of polymer produced by X15ld harboring pP4xylBC and pPs619C1437Pct540. GA, LA and 2HB indicates glycolate, d‐lactate and d‐2‐hydroxybutyrate, respectively.
**Fig. S4.** Fed‐batch cultures of X15ld expressing XylBC_*ccs*_, PhaC1437 and Pct540. Time profiles of dry cell weight (DCW), concentration of xylose and metabolites (d‐lactic, glycolic and acetic acids, ethylene glycol and polymer) and polymer contents by X15ld harboring pPs619C1437Pct540 and (A) pTacxylBC, (B) pP1xylBC, (C) pP2xylBC, (D) pP3xylBC, (E) pP4xylBC and (F) pP5xylBC. (G) Polymer contents and compositions of produced polymers in X15ld harboring pP5xylBC and pPs619C1437Pct540. GA, EG, LA, AA and 2HB indicate glycolic acid, ethylene glycol, d‐lactic acid, acetic acid and d‐2‐hydroxybutyrate, respectively.
**Fig. S5.** Fed‐batch cultures of X17ld expressing XylBC_*ccs*_, PhaC1437 and Pct540. Time profiles of dry cell weight (DCW), concentration of xylose and metabolites (d‐lactic, glycolic and acetic acids, ethylene glycol and polymer) and polymer contents by X17ld harboring pPs619C1437Pct540 and (A) pP1xylBC, (B) pP2xylBC, (C) pP3xylBC, (D) pP4xylBC and (E) pP5xylBC. (F) X17ld harboring pPs619C1437Pct540 and pP5xylBC was cultured in l‐isoleucine supplemented medium. GA, EG, LA and AA indicate glycolic acid, ethylene glycol, d‐lactic acid and acetic acid, respectively.
**Fig. S6.** Live/dead assay of polymers produced by engineered *E. coli*. Human mesenchymal stem cells (hMSCs) were incubated after 4 days on (A) cover glass, (B) PLGA coated glass, (C) poly(d‐LA‐*co*‐GA‐*co*‐d‐2HB) coated glass. (live cells, green; dead cells, red).
