# Supplementary material for: Engineering the xylose‐catabolizing Dahms pathway for production of poly(d‐lactate‐co‐glycolate) and poly(d‐lactate‐co‐glycolate‐co‐d‐2‐hydroxybutyrate) in Escherichia coli
Source: Microb Biotechnol. 2017 Apr 19;10(6):1353–64. doi: 10.1111/1751-7915.12721 (PMC5658605; doi:10.1111/1751-7915.12721)
Supplement: Supplementary file 1 — Table S1. Primers used in this study. Fig. S1. In silico genome‐scale analysis of the cell maximum growth rate changes caused by introducing Dahms pathway. Fig. S2. Polymer contents and compositions of polymers produced by X15lda strain harboring pPs619C1437Pct540 and different XylBCccs expression vectors. Fig. S3. NMR analysis. (A) 1H NMR spectrum and (B) 13C NMR spectrum of polymer produced by X15ld harboring pP4xylBC and pPs619C1437Pct540. GA, LA and 2HB indicates glycolate, d‐lactate and d‐2‐hydroxybutyrate, respectively. Fig. S4. Fed‐batch cultures of X15ld expressing XylBCccs, PhaC1437 and Pct540. Time profiles of dry cell weight (DCW), concentration of xylose and metabolites (d‐lactic, glycolic and acetic acids, ethylene glycol and polymer) and polymer contents by X15ld harboring pPs619C1437Pct540 and (A) pTacxylBC, (B) pP1xylBC, (C) pP2xylBC, (D) pP3xylBC, (E) pP4xylBC and (F) pP5xylBC. (G) Polymer contents and compositions of produced polymers in X15ld harboring pP5xylBC and pPs619C1437Pct540. GA, EG, LA, AA and 2HB indicate glycolic acid, ethylene glycol, d‐lactic acid, acetic acid and d‐2‐hydroxybutyrate, respectively. Fig. S5. Fed‐batch cultures of X17ld expressing XylBCccs, PhaC1437 and Pct540. Time profiles of dry cell weight (DCW), concentration of xylose and metabolites (d‐lactic, glycolic and acetic acids, ethylene glycol and polymer) and polymer contents by X17ld harboring pPs619C1437Pct540 and (A) pP1xylBC, (B) pP2xylBC, (C) pP3xylBC, (D) pP4xylBC and (E) pP5xylBC. (F) X17ld harboring pPs619C1437Pct540 and pP5xylBC was cultured in l‐isoleucine supplemented medium. GA, EG, LA and AA indicate glycolic acid, ethylene glycol, d‐lactic acid and acetic acid, respectively. Fig. S6. Live/dead assay of polymers produced by engineered E. coli. Human mesenchymal stem cells (hMSCs) were incubated after 4 days on (A) cover glass, (B) PLGA coated glass, (C) poly(d‐LA‐co‐GA‐co‐d‐2HB) coated glass. (live cells, green; dead cells, red). [file MBT2-10-1353-s001.docx]

**Table S1.** Primers used in this study.

| **Primer name** | **Sequences** |
| --- | --- |
| xylAB_F | atgcaagcctattttgaccag |
| xylAB_R | aaaatcttctctcatccgccaaaacagccaagcttttacgccattaatggcagaag |
| tac_xylAB_F | ccggccaaccccagcatgaggtccgccttgtctaattgacaattaatcatcggctcg |
| tac_xylAB_R | cgaacgcgatcgagctggtcaaaataggcttgcattgtttcctgtgtgaaattgttatc |
| 100_F | ctaggtacagtgctagc tgtggaattgtgagcggataac |
| 100_R | gactgagctagccgtcaa cagctcatttcagaatatttgcc |
| 101_F | cctaggtattatgctagc tgtggaattgtgagcggataac |
| 101_R | actgagctagctgtaaa cagctcatttcagaatatttgcc |
| 118_F | tcctaggtattgtgctagc tgtggaattgtgagcggataac |
| 118_R | ctgagctagccgtcaa cagctcatttcagaatatttgcc |
| 105_F | cctaggtactatgctagc tgtggaattgtgagcggataac |
| 105_R | actgagctagccgtaaa cagctcatttcagaatatttgcc |
| 117_F | ctagggattgtgctagc tgtggaattgtgagcggataac |
| 117_R | gactgagctagctgtcaa cagctcatttcagaatatttgcc |

**
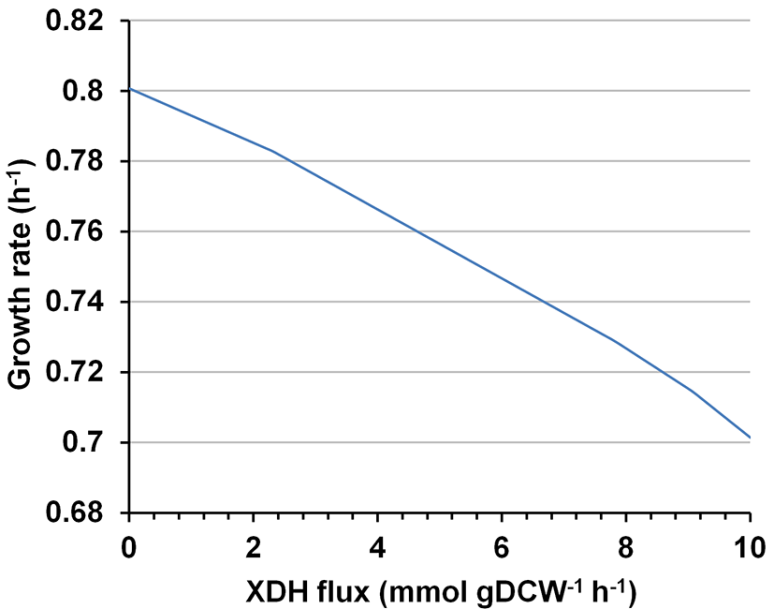
**

**Fig. S1.** *In silico* genome-scale analysis of the cell maximum growth rate changes caused by introducing Dahms pathway.

**
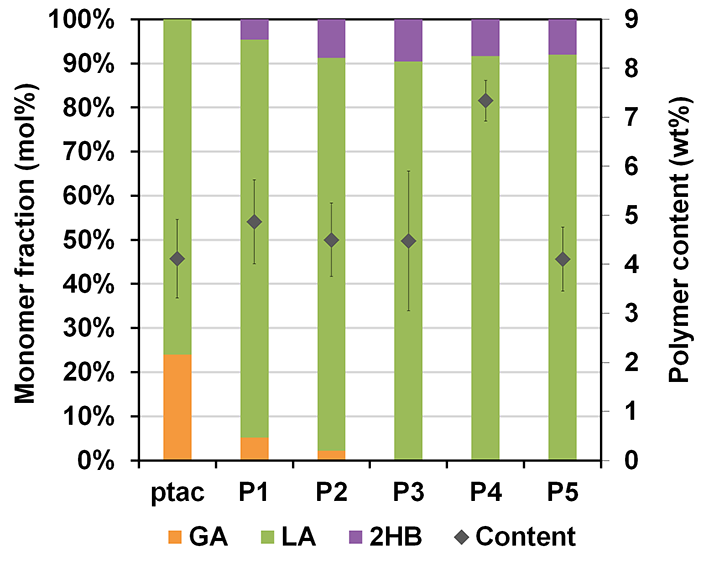
**

**Fig. S2.** Polymer contents and compositions of polymers produced by X15lda strain harboring pPs619C1437Pct540 and different XylBC*_ccs_* expression vectors.

**
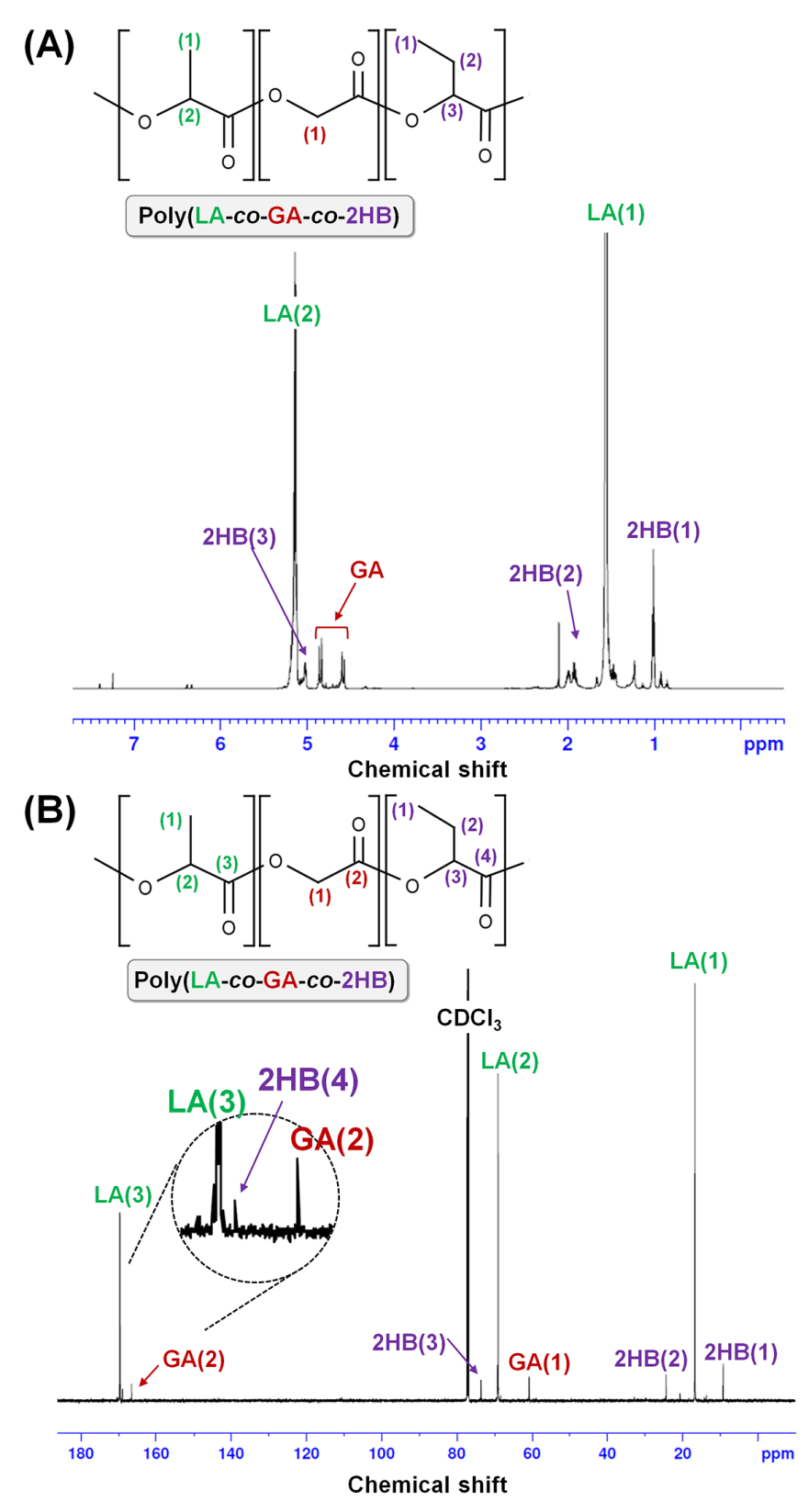
**

**Fig. S3.** NMR analysis. (A) ^1^H NMR spectrum and (B) ^13^C NMR spectrum of polymer produced by X15ld harboring pP4xylBC and pPs619C1437Pct540. GA, LA and 2HB indicates glycolate, d-lactate and d-2-hydroxybutyrate, respectively.








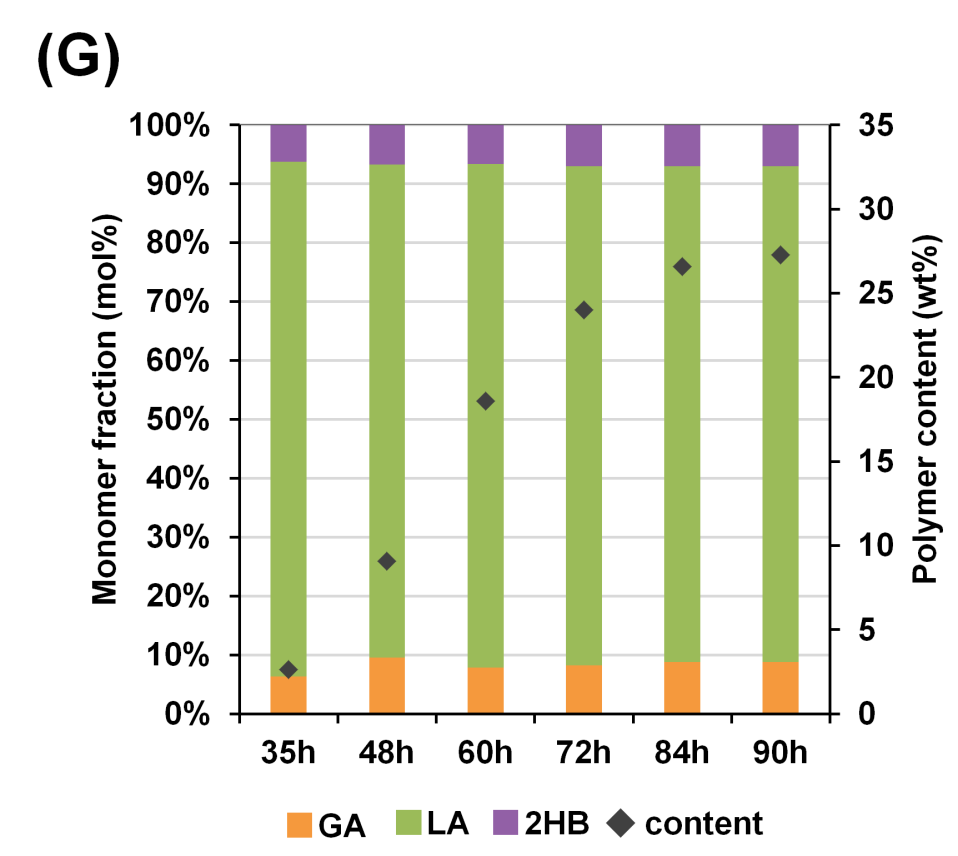


**(B)**

**(A)**

**(D)**

**(C)**

**(F)**

**(E)**

**Fig. S4. Fed-batch cultures of X15ld expressing XylBC*_ccs_*, PhaC1437 and Pct540.** Time profiles of dry cell weight (DCW), concentration of xylose and metabolites (d-lactic, glycolic and acetic acids, ethylene glycol and polymer) and polymer contents by X15ld harboring pPs619C1437Pct540 and (A) pTacxylBC, (B) pP1xylBC, (C) pP2xylBC, (D) pP3xylBC, (E) pP4xylBC and (F) pP5xylBC. (G) Polymer contents and compositions of produced polymers in X15ld harboring pP5xylBC and pPs619C1437Pct540. GA, EG, LA, AA and 2HB indicate glycolic acid, ethylene glycol, d-lactic acid, acetic acid and d-2-hydroxybutyric acid, respectively.

**(G)**

**





**

**(B)**

**(A)**

**(D)**

**(C)**

**(F)**

**(E)**

**Fig. S5. Fed-batch cultures of X17ld expressing XylBC*_ccs_*, PhaC1437 and Pct540.** Time profiles of dry cell weight (DCW), concentration of xylose and metabolites (d-lactic, glycolic and acetic acids, ethylene glycol and polymer) and polymer contents by X17ld harboring pPs619C1437Pct540 and (A) pP1xylBC, (B) pP2xylBC, (C) pP3xylBC, (D) pP4xylBC and (E) pP5xylBC. (F) X17ld harboring pPs619C1437Pct540 and pP5xylBC was cultured in l-isoleucine supplemented medium. GA, EG, LA and AA indicate glycolic acid, ethylene glycol, d-lactic acid and acetic acid, respectively.

**
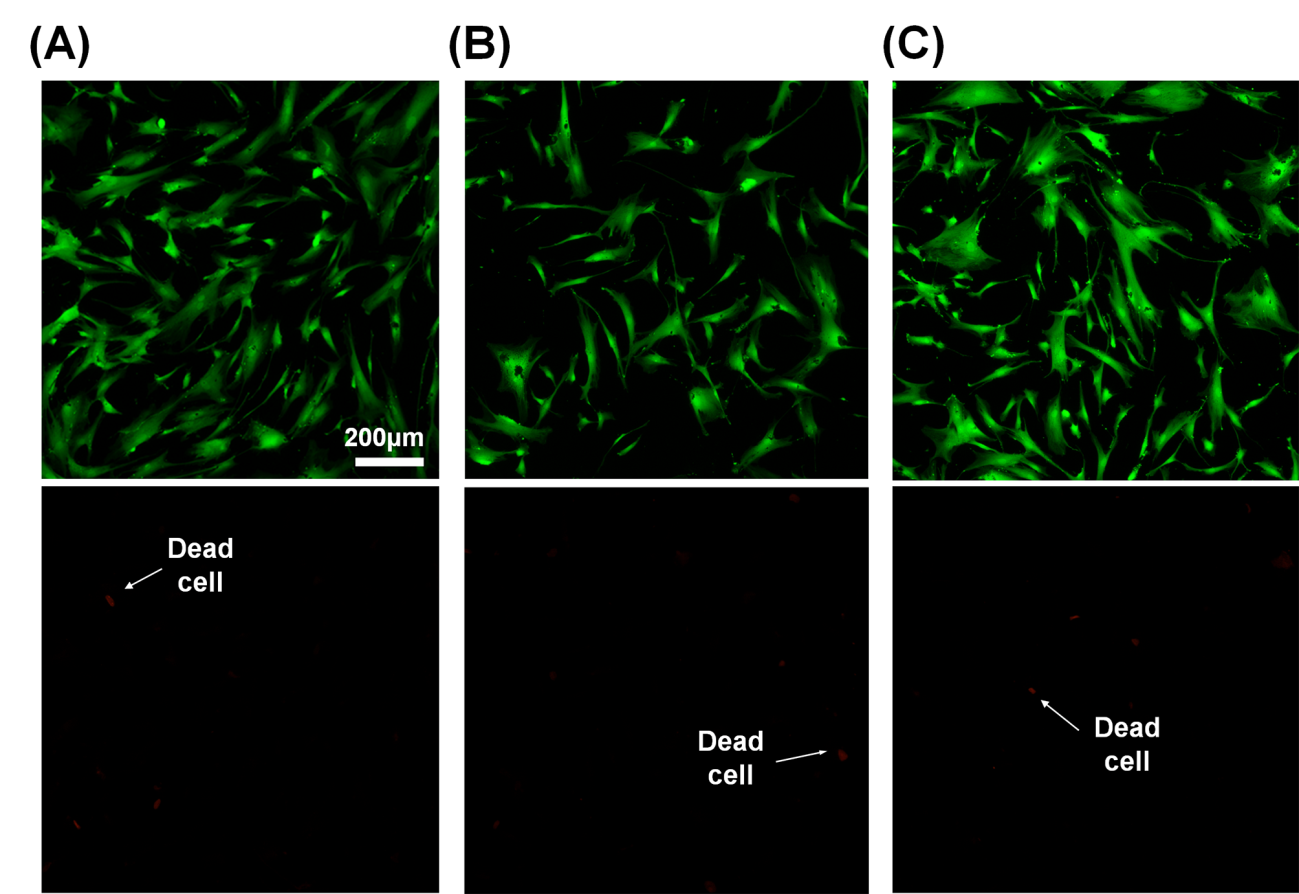
**

**Fig. S6.** Live/dead assay of polymers produced by engineered *E. coli*. Human mesenchymal stem cells (hMSCs) were incubated after 4 days on (A) cover glass, (B) PLGA coated glass, (C) poly(d-LA-*co*-GA-*co*-d-2HB) coated glass. (live cells, green; dead cells, red).
